# Automated surveillance of antimicrobial consumption in intensive care, northern Sweden: an observational case study

**DOI:** 10.1186/s13756-024-01424-2

**Published:** 2024-06-18

**Authors:** Andreas Winroth, Mattias Andersson, Peter Fjällström, Anders F. Johansson, Alicia Lind

**Affiliations:** 1 Department of Clinical Microbiology, Umeå University, SE-90187 Umeå, Sweden; 2 Center for Intensive Care (IT unit), Norrlands universitetssjukhus, 90185 Umeå, SE Sweden; 3 Department of Diagnostics and Intervention, Umeå University, SE-90187 Umeå, Sweden; 4 Department of Infection Prevention and Control Region Västerbotten, Norrlands universitetssjukhus, SE-90185 Umeå, Sweden

**Keywords:** Automated surveillance, Intensive care, Antibiotics, Antimicrobial consumption, Antimicrobial stewardship, Antimicrobial resistance

## Abstract

**Background:**

The digitalization of information systems allows automatic measurement of antimicrobial consumption (AMC), helping address antibiotic resistance from inappropriate drug use without compromising patient safety.

**Objectives:**

Describe and characterize a new automated AMC surveillance service for intensive care units (ICUs), with data stratified by referral clinic and linked with individual patient risk factors, disease severity, and mortality.

**Methods:**

An automated service collecting data from the electronic medical record was developed, implemented, and validated in a healthcare region in northern Sweden. We performed an observational study from January 1, 2018, to December 31, 2021, encompassing general ICU care for all ≥18-years-olds in a catchment population of 270000 in secondary care and 900000 in tertiary care. We used descriptive analyses to associate ICU population characteristics with AMC outcomes over time, including days of therapy (DOT), length of therapy, defined daily doses, and mortality.

**Results:**

There were 5608 admissions among 5190 patients with a median age of 65 (IQR 48-75) years, 41.2% females. The 30-day mortality was 18.3%. Total AMC was 1177 DOTs in secondary and 1261 DOTs per 1000 patient days and tertiary care. AMC varied significantly among referral clinics, with the highest total among 810 general surgery admissions in tertiary care at 1486 DOTs per 1000 patient days. Case-mix effects on the AMC were apparent during COVID-19 waves highlighting the need to account for case-mix. Patients exposed to more than three antimicrobial drug classes (*N* = 242) had a 30-day mortality rate of 40.6%, with significant variability in their expected rates based on admission scores.

**Conclusion:**

We introduce a new service and instructions for automating local ICU-AMC data collection. The versatile long-term ICU-AMC metrics presented, covering patient factors, referral clinics and mortality outcomes, are expected to be beneficial in refining antimicrobial drug use.

**Supplementary Information:**

The online version contains supplementary material available at 10.1186/s13756-024-01424-2.

## Background

Infections caused by resistant bacteria pose a major threat to patient safety in intensive care units (ICUs) [1, 2]. The strongest driver of antimicrobial resistance (AMR) is misuse or overuse of antimicrobials [3]. Intensive care inherently involves a substantial use of antimicrobials; about 70% of all ICU patients receive antibiotics during their ICU admission, while 50% have a suspected or proven infection [4]. Antimicrobial stewardship is a coherent set of actions aimed at structurally improving and reducing antimicrobial use without exposing the patient to a risk of undertreatment [5–7]. A prerequisite for antimicrobial stewardship programs (ASPs) in intensive care is reliable measures of antimicrobial consumption (AMC) with sufficient resolution to perceive changes in overall use and consumption patterns over time [8]. Mortality rates should be monitored closely in a standardised manner to ensure that a reduction in antimicrobial use does not compromise the risk of mortality [9].

The rapid digitalisation of information systems in intensive care offers opportunities to measure antimicrobial use and consumption in an automated and uniform manner. These data can be readily integrated with other patient information, including the identification of risk factors for infections, such as the use of mechanical ventilation, central venous catheters, and renal replacement therapy, and further information on individual patients´ predictive mortality scores and ICU mortality.

In this study, we describe and characterise a new automated surveillance service designed to perform real-time monitoring of AMC in the ICU by the three metrics days of therapy (DOT), length of therapy (LOT), and defined daily dose (DDD). We provide a detailed description of the surveillance service and source code for data extraction to facilitate local integration of the automated service presented here in other ICU settings.

## Methods

### Study design and clinical setting

This was an observational case study of a new automated AMC surveillance service run at general ICUs of the public healthcare provider Region Västerbotten in northern Sweden. The ICUs under study operate in a low AMC setting, with a national average of 10.1 DDD of systemic antimicrobials per 1000 inhabitants per day in community and hospital sectors which compares to the EU/EEA average of 16.4 DDD [10]. Sweden also has a low number of ICU beds, with 5.1 beds per 100000 population [11]. The new automated AMC surveillance service was implemented at three general ICUs located at Norrlands universitetssjukhus (a 400-bed tertiary care medical teaching hospital with 10 ICU beds), at Skelleftea hospital (a 180-bed secondary care hospital with 7 ICU beds), and at Lycksele hospital (70 bed secondary care hospital with 5 ICU beds). Norrlands universitetssjukhus is the referral hospital for highly specialised care with a source population of approximately 900000 people in the northern low-populated geographical half of Sweden. ICU admissions include critically ill medical, surgical, neurological, neurosurgical, and trauma patients of all ages. The ICUs at Skelleftea hospital, Lycksele hospital together with the ICU at Norrlands universitetssjukhus provide secondary care for a source population of 270000 living in Region Västerbotten, one of the four healthcare regions in the northern geographical half of Sweden.

The three ICUs were staffed by intensive care specialist physicians, residents of anaesthesiology, and nurses specialised in intensive care. Infectious disease consultants conducted daily reviews of patients' systemic antimicrobial therapy at the tertiary care hospital. At the secondary care hospitals, on-demand telephone service by infectious disease consultants was available. During the two periods of the COVID-19 pandemic, the number of ICU beds was temporarily increased at the tertiary care ICU. Between April 3rd and June 14th, 2020, and from February 8th to May 28th, 2021, the average numbers of available beds were 22 and 17, with occupancy rates of 42% and 67% respectively. The average number of tertiary care ICU beds outside these periods was 9.5 beds, with an occupancy rate of 75%.

### Data source and reporting tool

In 2016, a new electronic medical record (EMR) for intensive care was implemented in the ICUs to electronically handle clinical patient monitoring data, medication prescriptions, and medication administration recordings [12]. In a collaborative effort between the Center for Intensive Care and the Department of Infection Prevention and Control in Region Västerbotten, a service for automated surveillance of AMC was developed and integrated with a new information service platform for managing and storing data generated by the new EMR. This is a Windows service that every hour imports key patient data from all ongoing admissions from the intensive care information system to an independent database. These data include digital registrations with timestamps of all drug doses with substance weight and route of administration for antimicrobials to provide metrics of AMC using a service from iMD Soft®, MetaVision Medication API. Active antimicrobial substances were classified according to the WHO anatomical therapeutic chemical classifications (ATC) 5^th^ level [13]. Timestamps represented the time recorded when an ICU nurse signed into the EMR to document that a dose had been administered to the patient. The AMC metrics of DOT and LOT were collected by a script identifying the time of the day´s first antimicrobial drug administration. Another script summarised the weight per drug administered per day and calculated the defined daily dose (DDD) using the WHO ATC/DDD Index 2023 [14]. By using an existing data flow for reporting data to the Swedish Intensive Care Registry (SIR) [15], AMC data at the individual patient level was linked with age, gender, length of stay (LOS), referring specialty, duration of mechanical ventilator treatment, duration of haemodialysis, duration of central venous catheter usage, simplified acute physiology score 3 (SAPS 3) at admission, ICU mortality rate, and 30-day mortality rate.

The surveillance service was developed in Microsoft Visual Studio 2022, mainly using the program language C# and Microsoft.NET version 4.6. The services are executed under Microsoft Internet Information Services (IIS, version 8) on a virtual server of the internal network, and the database is a Microsoft SQL server. A generic description of the surveillance service is available in Supplementary materials, Additional file 1, which is aimed at IT architects associated with units that want to integrate the service into their data storage system. The code used for digital data extraction is available, see the Availability of data and material section.

The validity of the service output data was confirmed through a reiterative process of logical checks and code revision until the output of the antimicrobial metrics matched the output of manual reviews of the EMR, which served as the gold standard. A total of 200 randomly sampled admissions were reviewed during this process. The accuracy and completeness of the AMC data was checked during the study period by yearly controls, including logical checks of output data from the surveillance service and manual medical record review of a random sample of 10 admissions.

**Fig. 1:**
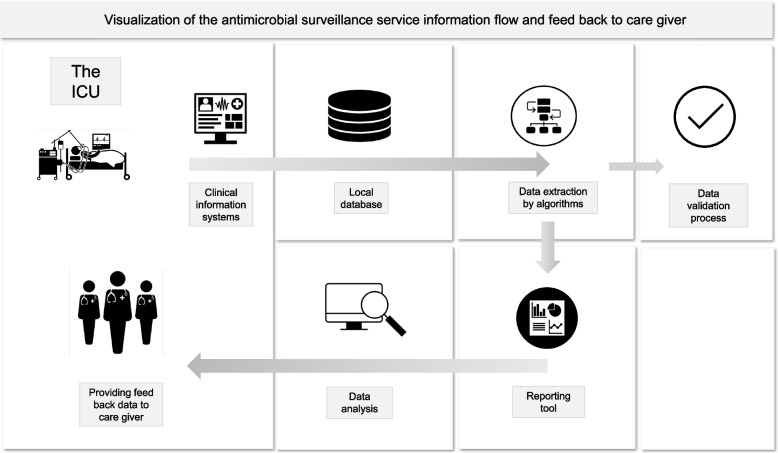
Real-time patient data is processed within a local database by algorithms that link AMC metrics with a comprehensive set of other relevant patient information. The reporting tool compiles data that can be utilised for analysis and facilitates the provision of feedback to caregivers

A reporting tool for caregivers was developed to compile patient information and generate standard reports in Excel format; see examples in Additional file 2. Figure 1 schematically illustrates the flow of information, culminating in the feedback provided to caregivers.

### Study population, data specifications and outcomes

All patients aged 18 years or older admitted to the three general ICUs between January 1, 2018, and December 31, 2021, were eligible and included. The following patient groups were not included as they were admitted to other specialized tertiary care ICUs: thoracic surgery patients and a subgroup of neurosurgery patients not in need of mechanical ventilation support.

**Fig. 2:**
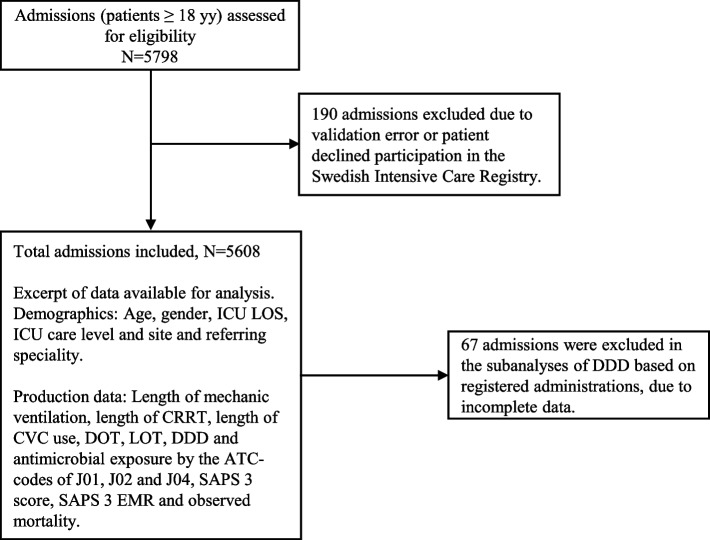
Flow chart detailing admissions, missing data, and an overview of the variables used for descriptive analyses

Electronic time stamps (date:hour:sec) were used to calculate the patient's duration of care in hours and antimicrobial treatment hours. In analyses using patient days as denominator, the duration of ICU care in hours was divided by 24. In cases of readmission within 48 hours, the subsequent admission was considered part of the previous intensive care episode. Transfers between the three ICUs were considered separate admissions to enable linkage of antimicrobial usage to the specific unit where antimicrobial administration took place (265 transfers from secondary care ICUs to the tertiary care ICU, and 127 in the opposite direction). Due to the small total number of admissions (*N*=431) and similar patient populations at the two secondary care ICUs, data from these units were aggregated before subsequent descriptive analyses. ICU patients in the secondary care ICUs who were temporarily provided care in a postanaesthetic care unit (370 admissions) were included. Details on study design and the population included and excluded is shown in Fig. 2.

AMC was normalised per 1000 patient days across all metrics to control for differences in how many patients are hospitalized each day. DOT was defined as the number of days that a patient received an antibacterial or an antifungal drug regardless of the dose (i.e., a patient receiving several antimicrobial agents the same day will have more than one DOT per day). LOT was defined as the number of days a patient received antimicrobial agents, irrespective of the number of different antimicrobials. DDD was calculated utilising the automatically collected administrated drug substance weights and the WHO ATC/DDD Index 2023 [14]. For comparison, pharmacy DDD data were also obtained from the Pharmacy Centre of Region Västerbotten, a measure representing all the antimicrobials supplied to the medicine shelf at the ICUs. To reduce bias in comparisons between the DDDs based on the administered doses per patient and the DDDs supplied to the medicine shelf where the latter may include patients <18-year-old, the number of admissions with <18-year-olds was accounted for (*N*=787).

Individual antimicrobial agents as well as antimicrobial classes were considered in the analyses using AMC metrics. In addition to metrics per 1000 patient days, LOT per admission, average LOT per antimicrobial use admission (i.e., average LOT in admissions where patients were administered an antimicrobial), and DOT/LOT ratios (i.e., a measure of frequency of exposure to more than one antimicrobial simultaneously), were used in our analyses. Finally, a metric denoting antimicrobial exposure was analysed. Antimicrobial exposure at the ICU was defined at five levels as follows: no administration of antimicrobials during patient admission; administration of one, two, three, or more than three different antimicrobial drug classes. Antimicrobial exposure was separately analysed for ICU stays >48 hours or <48 hours under the assumption that longer stays may represent more severe disease and a higher risk of healthcare-associated infection (HAI) [16] requiring more antimicrobial drug use. In addition, the antimicrobial exposure variable was investigated in relation to predicted and observed ICU mortality using the SAPS 3 EMR as a predictor of mortality.

### Missing data

A total of 190 admissions (3%) were excluded from the study due to validation errors in the database or patient refusal to participate in the Swedish Intensive Care Registry (SIR). ATC-code linkage was missing to the DOT variable in 67 admissions, equivalent to 132 DOT (6‰) out of 22788 DOT registered during the period. DDD data for sulfamethoxazole/trimethoprim was missing due to not having a defined DDD and DDD for colistin for dosing being measured in million units (MU), a unit that could not be processed by the automated surveillance service. The two drugs missing DDD values accounted for 2.8% of the total antibacterial consumption during the study period as measured by the DOT metric. Missing values were handled by omission in analyses, imputation of missing data was not performed.

### Statistics and graphing

We used a descriptive strategy to characterize the new AMC surveillance service and associate the ICU population characteristics with AMC outcomes and mortality. The results are presented as numerical values, or percentages. Medians with the interquartile ranges (IQRs) are used where applicable. We characterized and described changes in AMC over time in relation to case-mix by plotting DOT per calendar day in relation to two major COVID-19 waves during the pandemic that substantially changed the case-mix at the ICUs. We used a general additive model with cubic splines and 8 knots to estimate and visualize the trend of the response variable DOT per 1000 patient days DOT over calendar time [17]. Data processing was carried out by R version 4.2.2 (2022-10-31 ucrt) and RStudio 2023.09.1+494 "Desert Sunflower". The R-packages used was Tidyverse 1.3.1, Lubridate 1.8.0, and ggplot2 3.3.6. Affinity Designer 2, version 2.3.0, was used for finalising the figures.

## Results

### Patients and mortality

Between January 1, 2018, and December 31, 2021, a total of 5608 ICU admissions were recorded among 5190 patients aged 18 years and older (Table 1). Of these admissions, 2864 were in the tertiary care ICU and 2744 were in the secondary care ICUs. There was an overrepresentation of men in both tertiary and secondary care.Patients aged 60-79 years accounted for 52% and 44% of the total patient population in the tertiary and secondary care ICUs, respectively. Both the estimated 30-day mortality rates and the observed mortality rates were higher in the tertiary ICU.

**Table 1:** Patient characteristics by ICU care level

Characteristic	All ICUs (*N*=5608)	Secondary care ICUs (*N*=2744)	Tertiary care ICU (*N*=2864)
Median age, (IQR)-yr	65 (48-75)	66 (45-76)	65 (51-74)
Female sex -no. (%)	2315 (41.3)	1156 (42.1)	1159 (40.5)
Admissions, LOS > 48 h -no.	2050	775	1275
Patient days – no.	18537	7068	11469
LOS in days, median (IQR)	1.2 (0.6-3.4)	1.0 (0.5-2.4)	1.8 (0.7-4.8)
Admissions with IMV -no. (%)	2205 (39.3)	566 (20.7)	1639 (57.2)
IMV hours, median (IQR)^a^	47 (11-154)	28 (4-119)	54 (14-161)
Admissions with CRRT -no. (%)	153 (2.7)	55 (2.0)	98 (3.4)
CRRT hours, median (IQR)^b^	76 (36-153)	70 (25-140)	89 (40-177)
CVC hours, median (IQR)^c^	75 (29-178)	56 (22-143)	86 (38-195)
SAPS 3 score, median (IQR)	53 (43-64)	51 (41-61)	56 (46-76)
SAPS EMR, median (IQR)	0.10 (0.03-0.26)	0.08 (0.02-0.21)	0.14 (0.05-0.32)
ICU mortality, %	8.0	6.5	9.4
30 days mortality, %	18.3	17.7	19.0

*IQR* Interquartile range (25^th^ -75^th^ percentile), *LOS* Length of stay in patient days, IMV Invasive mechanical ventilation, *CRRT* Continuous renal replacement therapy, CVC Central venous catheter, *SAPS 3 score* Simplified acute physiology admission score, *SAPS EMR* SAPS estimated mortality rate

^a^Among patients with IMV

^b^Among patients with CRRT

^c^Among patients with CVC use

The most common referral was from internal medicine (including cardiology), accounting for 44% of all ICU admissions and 34% of total ICU admissions and total ICU patient days, respectively, followed by general surgery (including vascular surgery and urology), accounting for 26% of both total ICU admissions and ICU patient days. Table 2 provides information on patients categorised by referring clinics and ICU care level.

**Table 2:** Admission characteristics by referring clinics and ICU care levels

**ICU level and clinic**	**Admissions -no. (women)**	**Patient days -no. (women)**	**Median LOS, days (IQR)**
Secondary care ICUs, referring clinic			
Internal medicine	1883 (784)	4962 (1865)	1.0 (0.5-2.4)
General surgery	647 (250)	1697 (562)	1.0 (0.5-2.6)
Orthopedic surgery	65 (28)	180 (62)	1.3 (0.5-3.2)
Other	149 (92)	229 (76)	0.6 (0.4-1.0)
Tertiary care ICU, referring clinic			
General surgery	810 (265)	3195 (952)	1.8 (0.9-4.6)
Internal medicine	594 (265)	1318 (490)	0.9 (0.4-2.1)
Neurosurgery	516 (245)	2701 (1311)	2.9 (0.9-7.9)
Infectious diseases	305 (112)	1974 (633)	3.5 (1.3-8.7)
Neurology	243 (93)	770 (249)	1.3 (0.6-3.9)
Orthopedic surgery	149 (48)	814 (196)	2.2 (1.1-5.9)
Oncology and haematology	64 (34)	272 (120)	2.0 (1.0-4.0)
Other	183 (98)	425 (188)	1.0 (0.6-2.5)

### Antimicrobial exposure by length of stay

Patients were exposed to one antimicrobial class or more in 66% (1864/2864) and in 47% (1296/2744) of all ICU admissions in the tertiary and secondary care ICUs, respectively. Among patients with an admission with a LOS exceeding 48 hours, 90% (1153/1275) and 84% (649/775) were exposed to at least one antimicrobial class in the respective ICU site. Exposure to a cephalosporin (2^nd^ or 3^rd^ generation) was the most frequent drug class exposure in both tertiary and secondary care hospitals, accounting for 37% (1073/2864) and 21% (575/2744), respectively, of all admissions. The most frequent antifungal exposure was to echinocandins at 12% (153/1275) and 7% (57/775) of all ICU admissions with a LOS >48 hours at the tertiary and secondary care levels, respectively. More information on antimicrobial exposure by LOS and ICU care level is provided in Supplementary materials, Additional file 3.

### Antimicrobials by demography

In the tertiary care and secondary care ICUs, the average length of antimicrobial therapy, (LOT) per admission was 3.3 and 2.1 days, respectively. The average LOTs per antimicrobial use admission were 5.1 and 4.4 days, and the DOT/LOT ratios were 1.51 and 1.46, respectively.

Men had a higher AMC (1292 DOTs in tertiary care and 1232 DOTs in secondary care per 1000 patient days) than women (1206 and 1086 DOTs per 1000 patient days, respectively). Among the age groups, 70-79 years had the highest AMC rates, with 1302 DOTs per 1000 patient days and 1264 DOTs per 1000 patient days in tertiary and secondary care ICU levels, respectively. DOT per 1000 patient days by age group and gender are shown in Supplementary materials, Additional file 4.

**Fig. 3:**
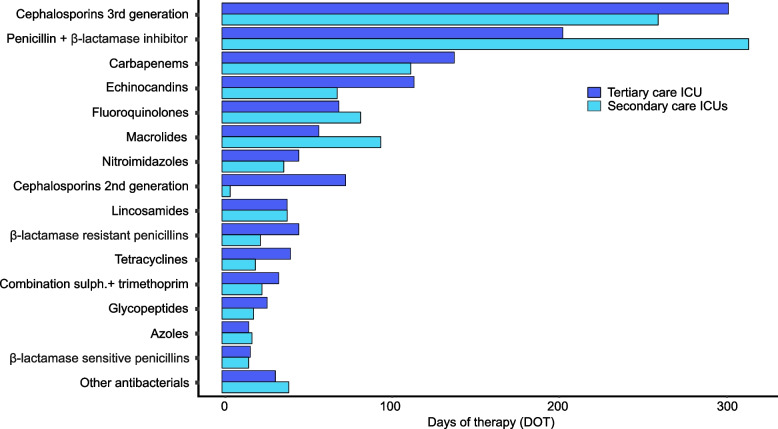
Days of therapy (DOT) by antimicrobial class during the years 2018-2021 in a tertiary care ICU (dark blue) and in secondary care ICUs (light blue)

Third-generation cephalosporins were the most commonly used antibacterial class in the tertiary care ICU, with a rate of 303 DOTs per 1000 patient days (27% of total antibacterial consumption in DOTs). In secondary care ICUs, the most frequently utilised class was penicillin combined with a beta-lactamase inhibitor (piperacillin with tazobactam), with a rate of 315 DOTs per 1000 patient days (29% of total antibacterial consumption). Figure 3 shows the number of DOTs by antimicrobial class and ICU care level from 2018-2021. The AMC per antimicrobial agent and DOT are provided in Additional file 5.

### Antimicrobial consumption by the referral clinic

**Fig. 4:**
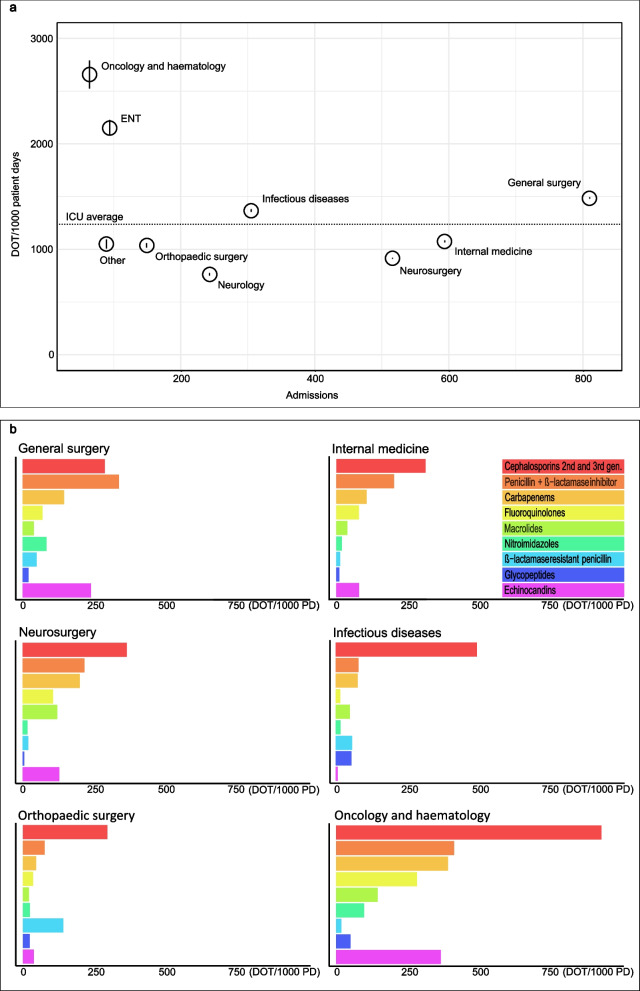
Panel A shows antimicrobial consumption data for the tertiary care ICU from 2018-2021. The dotted line represents the overall average, and the black open circles represent the data divided by the referral clinic. A vertical line through a black open circle is the 95% confidence interval for that clinic, and as should be expected, these intervals were barely visible for clinics with larger sample sizes (>200 admissions). Panel B shows more detailed antimicrobial consumption data divided by referring clinic and antimicrobial class by DOT per 1000 patient days

Throughout the study period, a total of 14466 DOTs were administered in the tertiary care ICU (1261 DOTs per 1000 patient days) and 8322 DOTs was administered in the two secondary care ICUs (1177 DOTs per 1000 patient days). Figure 4a shows the overall average DOT per 1000 patient days in the tertiary care ICU and the variation among different referral clinics. Hematological patients (64 admissions) had the highest AMC average with 2658 DOTs per 1000 patient days, while surgery patients, including vascular surgery and urology (810 admissions), represented the by far largest number of admissions, with a high average consumption of 1486 DOTs per 1000 patient days. In contrast, neurosurgery and internal medicine admissions had lower AMCs. Figure 4b shows a more detailed analysis of the data output from the surveillance service, including drug class consumption patterns per referring clinic to characterize the consumption per medical specialty.

### Comparison of different antimicrobial consumption metrics

The averages of the three different AMC metrics in the total ICU population from 2018 – 2021 were 1181 DOTs per 1000 patient days, 1161 DDD administered per 1000 patient days, and 1494 DDD according to pharmacy dispensing data per 1000 patient days. For certain drugs, like the beta-lactamase resistant penicillin cloxacillin, the difference between dispensing DDD, administered DDD, and the use measured by the DOT metric was substantial. Antimicrobial consumption over time by the three metrics are provided in Supplementary material, Additional file 6, illustrating the variation, especially in the pharmacy dispensing data. In Additional file 7, information on antimicrobial consumption described by metric and ICU care level is provided.

### Antimicrobial consumption in relation to case-mix

**Fig. 5:**
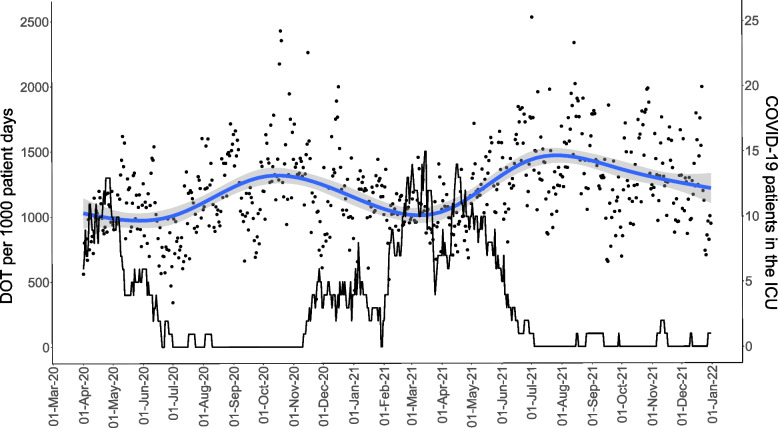
Relationships between case-mix change caused by COVID-19 pandemic waves (black line) and antimicrobial consumption measured in DOT per 1000 patient days (blue line with 95% confidence interval in grey). Black dots indicate daily antimicrobial use. Data is from three ICUs 1 April 2020 to 31 December 2021

To characterise the AMC over time and case-mix effects, consumption was plotted per calendar day over the COVID-19 pandemic waves (Fig. 5). The analysis showed a pattern of decreased consumption in periods with many COVID-19 patients admitted consistent with a more restricted antimicrobial use when the case-mix was changed.

### Antimicrobial exposure and mortality

**Fig. 6:**
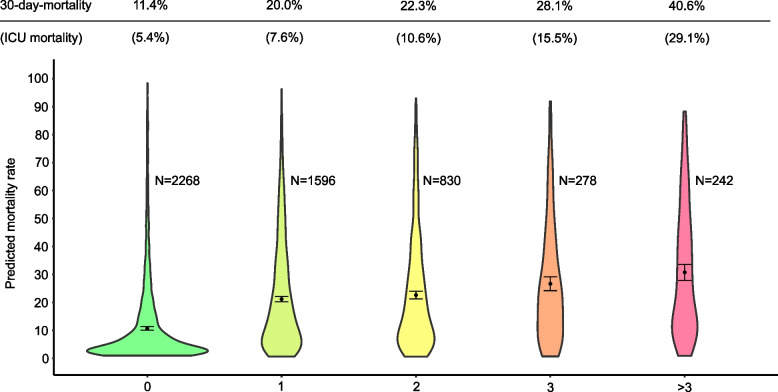
Relationships between the predicted mortality rate based on the SAPS 3 score at admission, observed 30-day mortality, and antimicrobial exposure during the ICU stay. Each density plot represents a category of antimicrobial exposure, from no exposure up to exposure to >3 antimicrobial drug classes. The observed 30-day mortality and ICU mortality are indicated above each plot. The number of patients in each category (N) and the average value (black dot with 95% confidence interval) are indicated

Characterizing different levels of antimicrobial exposure in the ICU in relation to observed and predicted 30-day mortality revealed a clear trend of more antimicrobial exposure being associated with higher mortality (Fig. 6). However, within each subgroup exposed to one or more antimicrobial classes, there was significant variability in the mortality predictions made at admission.

## Discussion

In this study, we describe and characterize an automated antimicrobial surveillance system for ICUs having healthcare data digitally available. We characterise the system's performance by presenting four-year prospectively registered AMC data along with automatically collected patient characteristics and production data. To facilitate local adoption of the surveillance service, a technical description and the source code for data export have been made available. With the extensive digitalisation of intensive care information systems, there has been a surge in interest regarding the automated monitoring of patient safety indicators, such as the AMC and healthcare-associated infections [18–20]. However, there is still a noticeable lack of publications describing the implementation phase and the characteristics of operative automated monitoring systems for AMC, including examples of how data could be compiled for conducting surveillance.

Our data registered by an automated surveillance system, reaffirms a high average AMC in an ICU setting caring for patients with high severity of illness. Importantly, the study also illustrates that antimicrobial consumption differs substantially by ICU care level and by different categories of the ICU population. In the tertiary care ICU, 66% of patients were exposed to at least one antimicrobial during their ICU stay, and 90% were exposed if the LOS exceeded 48 hours. These results resemble those of de Bus et al.'s four-year prospective registration of antimicrobial use in the Ghent University Hospital ICU, with exposure rates of 66% and 84%, respectively [21]. Additionally, they align with the findings from the EPIC III point-prevalence study, in which 70% of patients were receiving at least one antibiotic on the study day [4].

We identified large differences in consumption volumes and agent distributions when the ICU population was categorised by referring clinic. This implies that if the resolution and volume of surveillance data are sufficiently high, a referring clinic could serve as a valuable target unit when planning and directing ASPs in the ICU and suggests that a referring clinic should be considered when setting up automated surveillance.

We found that in our setting, the most used antimicrobial classes were 3^rd^ generation cephalosporins and penicillin with a beta-lactamase-inhibitor (piperacillin and tazobactam) in the tertiary care ICU and secondary care ICUs, respectively. In the only previous thorough evaluation of AMC in Swedish ICUs, published in 2022, Sjövall et al. reported that beta-lactamase-resistant penicillin (cloxacillin) was the most commonly used antibacterial class in Swedish ICUs [22]. The same class in our setting, measured by DOT, constituted only 4% and 2% of the total antibacterial consumption in the tertiary care ICU and secondary care ICUs, respectively. We think that this is an effect of the different ICU populations included and the use of pharmacy dispensing data in the study by Sjövall et al. The automated surveillance service used in this study excluded a large group of patients in perioperative care who received cloxacillin as a prophylactic antimicrobial drug. Cloxacillin is the prophylaxis of choice for high-volume surgeries such as orthopedic prosthetics and fracture surgery according to Swedish antimicrobial drug guidelines [23]. In many Swedish secondary care level hospitals, as in our setting, perioperative care is organised to share facilities with the ICU outside daytime hours, thereby allowing perioperative care patients access to the ICU medicine shelf. It was also apparent that the DDD metric overestimated cloxacillin consumption as compared with DOT, as the patients received a de facto higher daily dose than the cloxacillin ATC/DDD index [14]. The above example illustrates how different data sources and metrics of consumption may lead to disparate results. Similar patterns have previously been described by Polk et al. and Dalton et al. [24, 25] and in a comprehensive report by Kallen et al., who emphasise the importance of striving for uniform registration and data extraction procedures to attain accurate measurement of quantitative antimicrobial consumption [8].

Given the relatively low threshold for deploying automated monitoring of antimicrobial consumption, we believe it should be considered in all ICU settings with digitalised information systems. Monitoring automation would likely enhance both the reliability and reproducibility of surveillance data, thereby improving conditions for both intra- and interfacility comparisons, provided that the metrics and denominators are standardised.

There are several publications addressing questions about which measures, indicators, and outcomes are most appropriate for use in antimicrobial consumption surveillance and ASPs [26, 27]. For compelling reasons, days of therapy (DOT) has been recommended as a first-line AMC measure [28]. DOT is calculated from registered patient administrations and can therefore provide a measure with a resolution that makes data relevant both on an aggregated level and on a unit level in most settings. DDD calculated from the same data source may provide similar resolution, but the fixed nature of the metric is less adapted to the dynamics of intensive care, where dosing is frequently individualised based on the severity of illness, the infection site, and organ dysfunction. Additionally, the DDD metric faces challenges in accurately distinguishing the relative usage of different antimicrobial agents. This can lead to either an overestimation or an underestimation of the usage of specific compounds [24, 29]. Nevertheless, as the DDD metric is based on drug weight, it may provide valuable insights that DOT cannot offer, such as detecting alterations in dosing that might occur over time. To achieve the most comprehensive understanding of antimicrobial use, we agree with Stanic Benic et al., who argue in their systematic review on metrics for quantifying antimicrobial consumption that it is preferable to express consumption with at least two metrics simultaneously [30]. We suggest that both DOT and DDD, based on data from administered doses, should be included in the setup of automated surveillance for antimicrobial consumption.

We found an observed ICU mortality rate of 8.6% and a 30-day mortality rate of 18.3% in the entire ICU cohort. Patients exposed to more than three antimicrobial classes during their admission had an ICU mortality and 30-day mortality rates of 29.1% and 40.6%, respectively. There was an association between increasing predicted mortality and increasing antimicrobial exposure. The predicted and observed mortality agreed well for most patients with no exposure to antimicrobials. However, there was a clear pattern: in many patients exposed to antimicrobials, the predicted mortality based on SAPS 3 scores at admission provided underestimates of the observed mortality. Lindsey et al.´s systematic review on antimicrobial stewardship and ICU mortality [9] concluded that standardised estimates of mortality rates should be linked to antimicrobial use to ensure that patient outcomes are not compromised when implementing an ASP. This report serves as an example of how mortality monitoring could be used in conjunction with automated surveillance of antimicrobial consumption.

The limitations of the study include that the surveillance service was implemented in only one healthcare region in Sweden and within a single commercial intensive care information system. If implemented by multiple healthcare providers in multiple countries with different information systems, maybe challenges would have been revealed that we did not observe. Our results on antimicrobial metrics are setting-dependent and cannot be safely compared with ICU consumption in broader aspects, not even in the Swedish context, as consumption data reflect local prescribing patterns and patient mixes. However, this was not the main purpose of the study, which primarily aimed to characterize the new automated AMC service and provide examples of analyses and measures that may be useful for ASPs in ICU settings.

## Conclusions

Automated surveillance of antimicrobial consumption should be considered in all ICU settings with digitalised information systems. The DOT and DDD metrics are complementary and could both preferably be included in monitoring setups. Based on the findings of this observational case study of very different AMCs in different groups of ICU patients, it seems highly relevant to link mortality rates to antimicrobial consumption patterns to ensure that interventions aimed at changing antimicrobial use patterns do not compromise patient outcomes in subgroups of ICU patients.

### Supplementary Information

Additional file 1. 

Additional file 2. 

Additional file 3. 

Additional file 4. 

Additional file 5. 

Additional file 6. 

Additional file 7. 

## Data Availability

The code used for the extraction of digital data  from the ICU information system accessed by the automated surveillance system described  in the current work is available at GiTHub: https://github.com/johanssonresearch.

## Abbreviations

ICU: Intensive care unit
AMC: Antimicrobial consumption
DOT: Days of therapy
LOS: Length of stay
LOT: Length of therapy
DDD: Defined daily dose
ASP: Antimicrobial stewardship program
EMR: Electronic medical record
SIR: The Swedish intensive care registry
SAPS 3 score: Simplified acute physiology admission score
