# Supplementary material for: Automated surveillance of antimicrobial consumption in intensive care, northern Sweden: an observational case study
Source: Antimicrob Resist Infect Control. 2024 Jun 18;13:67. doi: 10.1186/s13756-024-01424-2 (PMC11186282; doi:10.1186/s13756-024-01424-2)
Supplement: Supplementary file 1 — Additional file 1. [file 13756_2024_1424_MOESM1_ESM.docx]

# **Additional file 1**

# ***Full description of the automated surveillance service of antimicrobial consumption.***

# *Antimicrobial report*

The antimicrobial report presents the antibiotic and antifungal usage per encounter for a specific department and a specific period, together with certain background data for each encounter. The result is displayed per encounter and consists of:

- Basic patient data
- Encounter data
- DOT (days of antibiotics therapy)
- LOT (length of antibiotics therapy)
- DDD (defined daily dose)
- Subdivision of Days of therapy per group of antibiotics and separate ATC codes
- Subdivision of amount of substance per group of antibiotics and separate ATC codes divided by the ATC/DDD index for each separate substance to calculate DDD.

The resulting data is presented on a web page and as an Excel file.

## *Prerequisites*

The EMR system for the intensive care unit is a Patient Data Management System called MetaVision from iMDsoft. It records patient data from medical technical units as well as all journal data from the intensive care, including ordered and administered medication. For each administered medication, the system records the medication, the amount, and the time. The system is used at all intensive care units in the county (five).

The MetaVision system should be set up according to the documentation with necessary departments, beds, and configurations to handle a patient flow at an ICU department. The antibiotics in use should be defined in the medication list, including their ATC code at fifth level.

The normal workflow for ordering and administering medication is assumed.

The following parameters are assumed to be defined and used:

- Birthdate (DateTime)
- Gender (Categorical text parameter)
- Home clinic (Categorical text parameter) – as defined by SIR^*)^
- Care level (Categorical text parameter) – as defined by SIR
- SAPS 3 Score (Numerical)
- SAPS 3 EMR – estimated mortality rate (Numerical)
- Ventilator mode (Free text)
- Workload score (Numerical) – VTS2014 score as defined by SIR
- Care result (Categorical text parameter)

*^*)^ SIR = the national Swedish Intensive Care Registry*

The ventilator mode should be recorded every minute that the ventilator is deployed. If it is not recorded, this parameter should not be used in calculating invasive ventilator time.

The following processes are assumed to be defined and used:

- Invasive ventilation
- Central venous catheter
- Continuous renal replacement therapy

## *Definitions*

DOT – days of therapy: Count one for each day the substance is administered. If more than one substance is ordered, count one per day and substance.

LOT – length of therapy: The number of days that any number of antibiotic substances were administered.

Amount of substance: The quantity of antibiotics substance administered (in mg, g or IE). Do not count dilution liquids or additional substances.

DDD – Defined Daily Dose: The DDD is the assumed average maintenance dose per day for a drug used for its main indication in adults (definition by WHO).

## *Algorithm*

### *Encounter selection*

The algorithm selects the encounters for the specified department that has a start time in the specified interval. Only encounters eligible for reporting to SIR (the Swedish Intensive Care Registry) are selected, i.e., encounters with care type IVA (intensive care) where the patient approves of participating in quality registers.

For the THIVA department (Thorax intensive care), both care levels IVA and TIVA are valid.

## *Data collection*

The source of the data collection is the MetaVision production database and data has been fetched using Standard query language, SQL. There are two databases, one for current production and one archive, and the encounters may be in either one (depending on the selected report time interval), thus both must be searched.

The full system contains numerous tables, but the following are of interest:

| **Table name** | **Contents** |
| --- | --- |
| Patients | One entry per encounter; contains civil registration number, name, current status, bed and department of the patient. |
| PatientLogs | Each patient has one entry for each status change and/or bed placement |
| Parameters | List of “parameters”, i.e., data point definitions customized by the user of the system. Contains all medications, all journal data definitions and process used in the patient care documentation. |
| DateTimeSignals | Stores parameter values of type DateTime |
| Signals | Stores parameter values of type Numeric or Boolean |
| TextSignals | Stores parameter values of type categorical (pre-defined text) |
| FreeTextSignals | Stores parameter values of type free text |
| RangeSignals | Stores parameter values of type medication and process |
| LogicalUnits | Department information |

To find all encounters from a certain department, we start by finding the department id in the LogicalUnits table. Then find the ID (PatientID) for all encounters that have an entry in the PatientLogs table for that department, in status IVA (2) and with a bed placement (BedID) in the specified time frame.

Proceeding from that list of PatientIDs, we will then find the admission date and discharge date for each patient. The admission date is found by finding the location time of the first entry in the PatientLogs table with status 2 and a bed ID. The discharge date is the first location time after the admission date where the ORStatus is 6 or 7 (discharged or archived), or when the department id changes to a different department.

| **Field** | **Comment** |
| --- | --- |
| Encounter Id | Internal Id |
| Period start | The time of admission to the ICU |
| Period end | The time of discharge from the ICU |
| Length of stay | Measured in hours |
| Birthdate | Parameter (DateTime) |
| Age | Calculated based on admission date and birthdate |
| Gender | Parameter (categorical) |
| Home clinic | Parameter (categorical) |
| Care level | Parameter (categorical) |
| SAPS 3 score | Parameter (numerical) |
| SAPS 3 EMR | Parameter (numerical) |
| Time with invasive ventilation | Time in hours (requires both an active process in MetaVision and a recorded input from the ventilator): Summarize the times from ventilator processes (RangeSignals). For the time to be valid, we also require that there are data from the ventilator during the same time (we use the ventilator mode parameter) |
| Time with central venous catheter | Time in hours. Summarize times from central venous catheter processes. |
| Time with continuous renal replacement therapy | Time in hours. Summarize times from CRRT processes. |
| Workload score | Total sum of workload scores according to the VTS 2014 system (SIR); these are stored per work shift as numerical parameters. |
| Care result | Alive or deceased – categorical parameter |
| 30-days mortality | Alive or deceased after 30 days. Mortality reports are fed back from SIR and stored in a separate database. Calculate 30-day mortality based on discharge date. |
| 90-days mortality | Alive or deceased after 90 days – same as above, but for 90 days. |
| Days of therapy | The sum of days where antibiotics have been administered to the patient, counting one day per antibiotic; for each day of the encounter, count the number of antibiotics administered. This is checked by finding entries in the RangeSignals table for the defined medications where the start or end time is on that day. |
| Length of therapy | The number of calendar days with any antibiotic therapy; count each day of the encounter where antibiotics has been administered. |

To collect days of therapy on a specific substance group, find all RangeSignals (valid, i.e., not marked as error) for the selected parameter IDs and patient ID and count the days covered.

To collect the amount of substance, go through the same RangeSignals and sum up the quantity (OriginalAmount or OriginalRate multiplied by time duration). The unit for the amount is defined in the medication parameter.

The DDD (Defined Daily Dose) is calculated by dividing the amount of substance with an index number from a table containing all relevant ATC codes and their DDD value. Indices were according to the ATC/DDD Index 2023 found on Relevant indices are found at the homepage of the WHO Collaborating Centre for Drug Statistics Methodology.

## *Antimicrobial substance groups*

Subdivision groups of antibiotics, based on ATC classification (for each group, measure DOT and substance amount respectively):

- J01DC+J01DD+J01DE Cephalosporins
- J01M Quinolones
- J01DH Carbapenems
- J01F Macrolides and lincosamides
- J01G Aminoglycosides
- J02A Antimycotics
- J01AA12 Tigecycline
- J01CA01 Ampicillin
- J01CE01 Benzylpenicillin
- J01CF02 Cloxacillin
- J01CR05 Piperacillin and enzyme inhibitors
- J01DC02 Cefuroxime
- J01DD01 Cefotaxime
- J01DD02 Ceftazidime
- J01DD04 Ceftriaxone
- J01DD52 Ceftazidime and beta-lactamase inhibitors
- J01DD54 Ceftriaxone and combinations
- J01DE01 Cefepime
- J01DF01 Aztreonam
- J01DH02 Meropenem
- J01DH03 Ertapenem
- J01DH51 Imipenem and cilastatin
- J01DH52 Meropenem and vaborbactam
- J01DH56 Imipenem, cilastatin and relebactam
- J01DI01 Ceftobiprole
- J01DI02 Ceftaroline
- J01DI04 Cefiderocol
- J01DI54 Ceftolozane and beta-lactamase inhibitors
- J01EE01 Sulfamethoxazole and trimethoprim
- J01FA01 Erythromycin
- J01FA10 Azithromycin
- J01FF01 Clindamycin
- J01GB01 Tobramycin
- J01GB03 Gentamicin
- J01GB06 Amikacin
- J01MA02 Ciprofloxacin
- J01MA12 Levofloxacin
- J01MA14 Moxifloxacin
- J01XA01 Vancomycin
- J01XA04 Dalbavancin
- J01XB01 Colistin
- J01XD01 Metronidazole
- J01XX01 Fosfomycin
- J01XX08 Linezolid
- J01XX09 Daptomycin
- J04AB02 Rifampicin
- J02AA01 Amphotericin B
- J02AC01 Fluconazole
- J02AC02 Itraconazole
- J02AC03 Voriconazole
- J02AC04 Posaconazole
- J02AC05 Isavukonazol
- J02AX04 Caspofungin
- J02AX06 Anidulafungin
