# Supplementary material for: Automated surveillance of antimicrobial consumption in intensive care, northern Sweden: an observational case study
Source: Antimicrob Resist Infect Control. 2024 Jun 18;13:67. doi: 10.1186/s13756-024-01424-2 (PMC11186282; doi:10.1186/s13756-024-01424-2)

# **Additional file 2**

# ***Screenshots of the web interface with examples of standard reports (text in Swedish).***


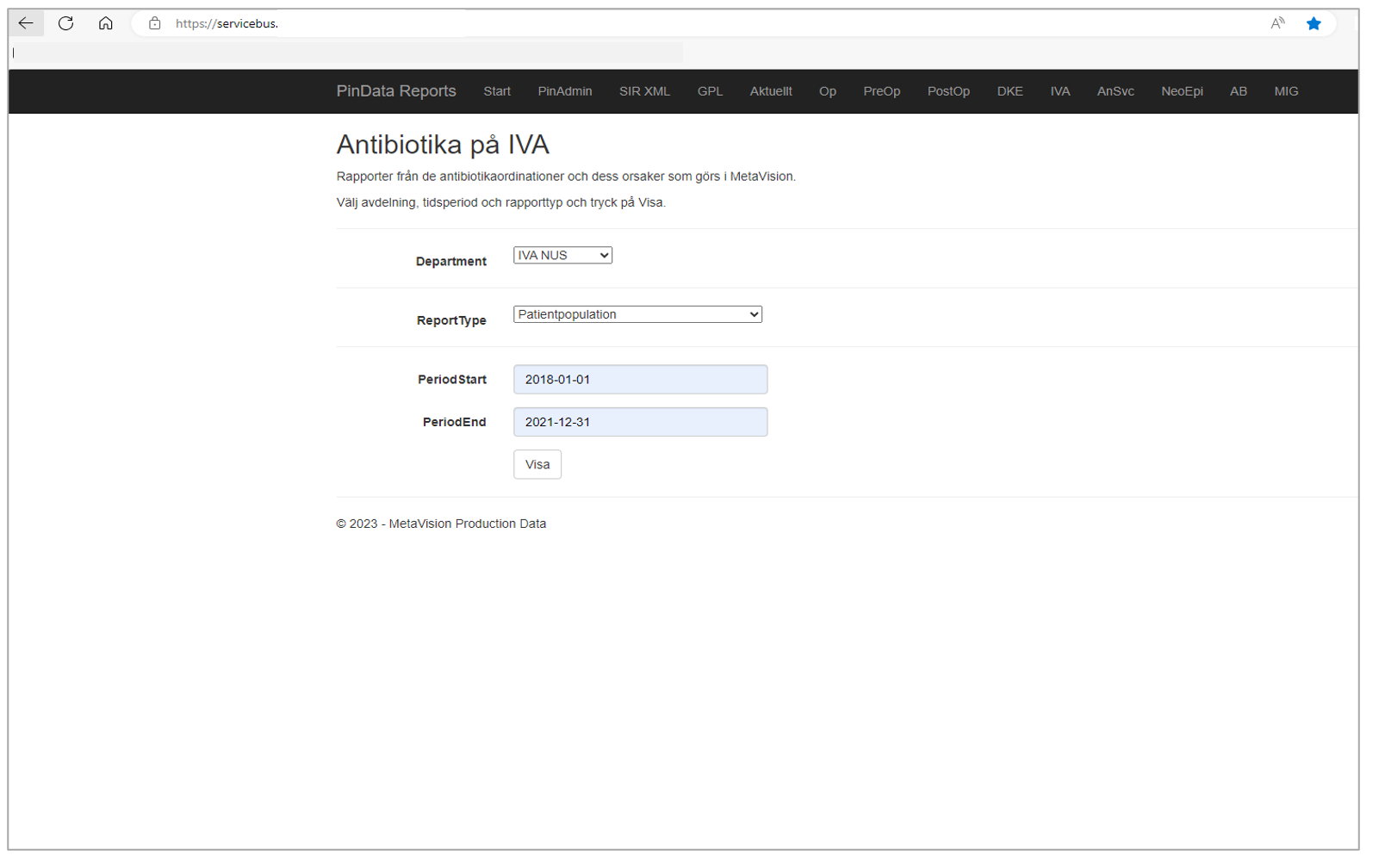


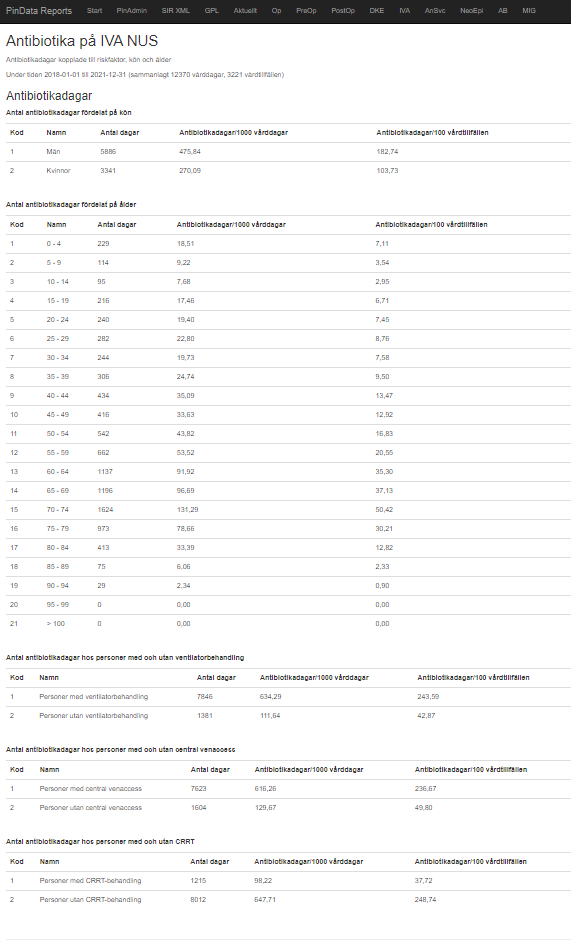


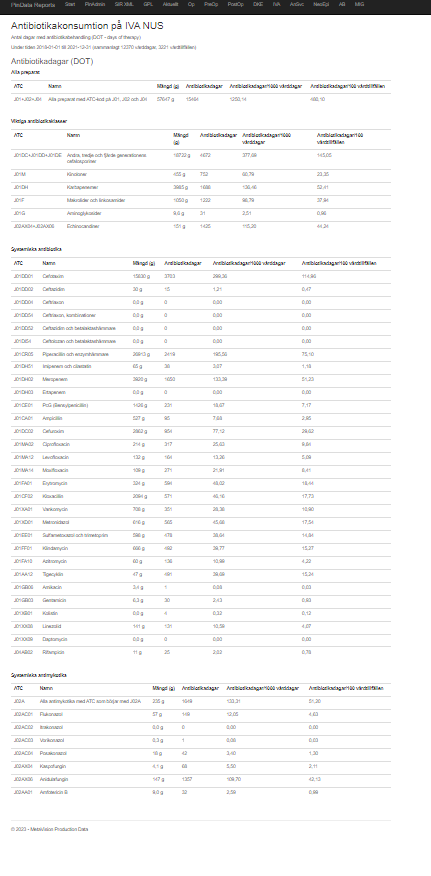

Supplement: Supplementary file 2 — Additional file 2. [file 13756_2024_1424_MOESM2_ESM.docx]
