# Supplementary material for: Automated surveillance of antimicrobial consumption in intensive care, northern Sweden: an observational case study
Source: Antimicrob Resist Infect Control. 2024 Jun 18;13:67. doi: 10.1186/s13756-024-01424-2 (PMC11186282; doi:10.1186/s13756-024-01424-2)
Supplement: Supplementary file 3 — Additional file 3. [file 13756_2024_1424_MOESM3_ESM.docx]

# **Additional file 3**

# ***Antimicrobial exposure by length of stay (LOS) and ICU care level 2018-2021.***


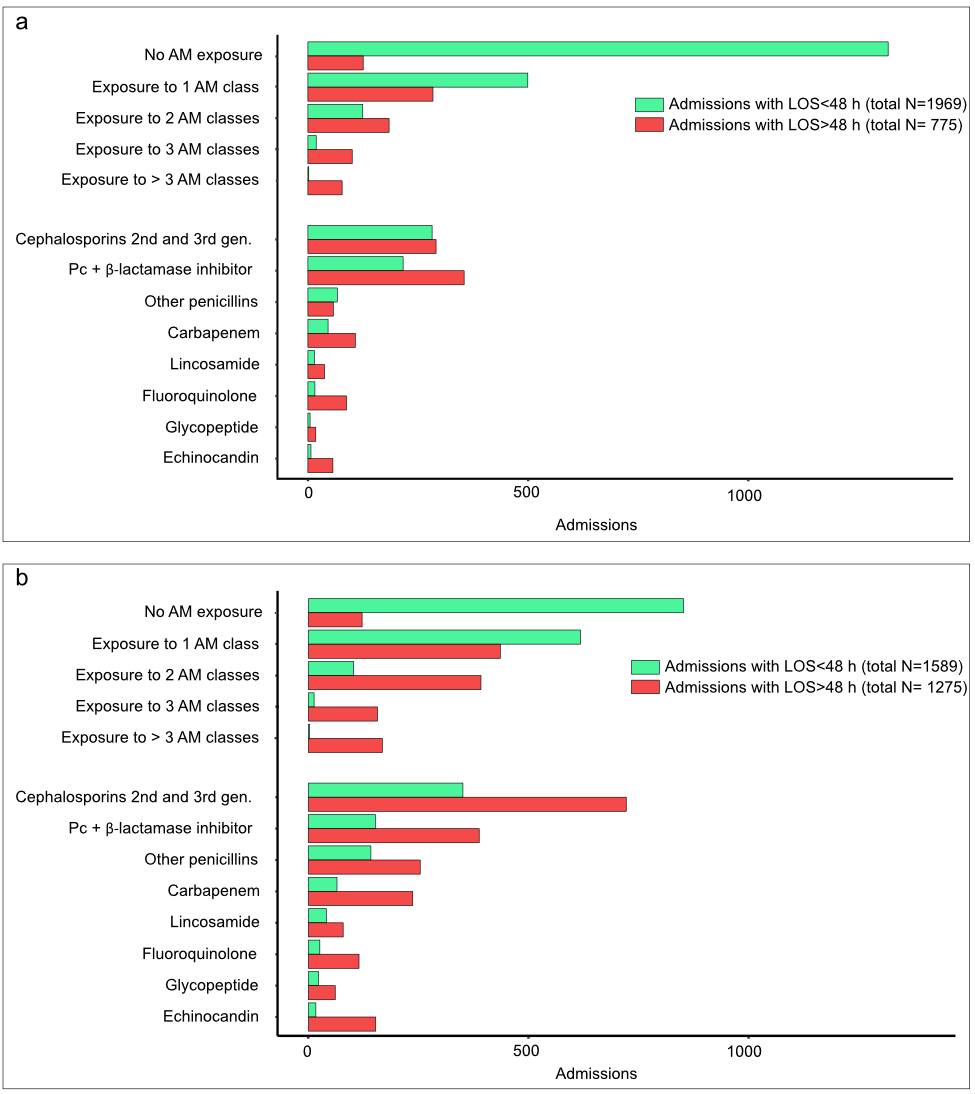


Additional file 3. Antimicrobial exposure by length of stay and ICU care level presented as number of admissions per type of exposure. Panel a) the secondary care ICUs and panel b) the tertiary care ICU.
