# Supplementary material for: Automated surveillance of antimicrobial consumption in intensive care, northern Sweden: an observational case study
Source: Antimicrob Resist Infect Control. 2024 Jun 18;13:67. doi: 10.1186/s13756-024-01424-2 (PMC11186282; doi:10.1186/s13756-024-01424-2)
Supplement: Supplementary file 4 — Additional file 4. [file 13756_2024_1424_MOESM4_ESM.docx]

# **Additional file 4**

# ***Antimicrobial consumption by gender, age group, and ICU care level 2018-2021.***


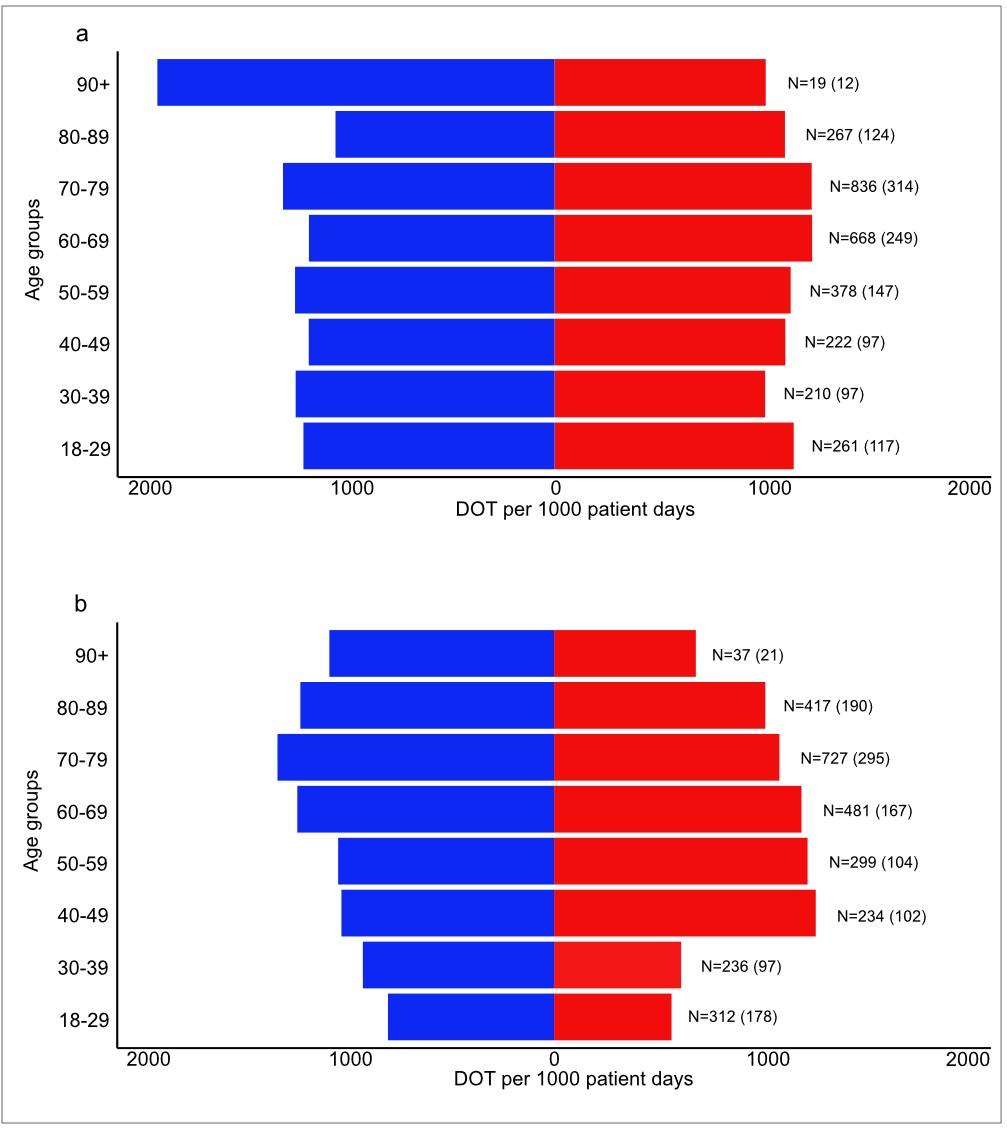


# Additional file 4. Antimicrobial consumption in DOT per 1000 patient days by age group and gender. Men are indicated by blue bars and women by red bars. Panel a shows data for the tertiary care ICU. Panel b shows aggregated data for the two secondary care ICUs. The total number of admissions per age group are indicated to the far right. Numbers in parentheses indicate admissions of women.
