# Supplementary material for: Automated surveillance of antimicrobial consumption in intensive care, northern Sweden: an observational case study
Source: Antimicrob Resist Infect Control. 2024 Jun 18;13:67. doi: 10.1186/s13756-024-01424-2 (PMC11186282; doi:10.1186/s13756-024-01424-2)
Supplement: Supplementary file 5 — Additional file 5. [file 13756_2024_1424_MOESM5_ESM.docx]

# **Additional file 5**

# ***Antimicrobial consumption by DOT, antimicrobial agent, and ICU care level 2018-2021.***

*Secondary care ICUs*

| **Antibacterial agent** | **DOT** | **DOT/ 1000 PD** | **Relative use (%)** |
| --- | --- | --- | --- |
| Cefotaxime | 1815 | 257 | 23.6 |
| Piperacillin and tazobactam | 2227 | 315 | 29.0 |
| Meropenem | 773 | 109 | 10.1 |
| Erythromycin | 587 | 83 | 7.6 |
| Moxifloxacin | 350 | 50 | 4.6 |
| Clindamycin | 275 | 39 | 3.6 |
| Metronidazole | 259 | 37 | 3.4 |
| Trimethoprim and sulph. | 171 | 24 | 2.2 |
| Cloxacillin | 162 | 23 | 2.1 |
| Tigecycline | 143 | 20 | 1.9 |
| Vancomycin | 133 | 19 | 1.7 |
| Ciprofloxacin | 127 | 18 | 1.7 |
| Penicillin G | 114 | 16 | 1.5 |
| Levofloxacin | 112 | 16 | 1.5 |
| Azitromycin | 81 | 11 | 1.1 |
| Gentamicin | 68 | 10 | 0.9 |
| Cefuroxime | 38 | 5 | 0.5 |
| Ampicillin | 32 | 5 | 0.4 |
| Ceftriaxone | 23 | 3 | 0.3 |
| Ertapenem | 15 | 2 | 0.2 |
| Imipenem and cilastatin | 11 | 2 | 0.1 |
| Ceftazidim and avibactam | 8 | 1 | 0.1 |
| Linezolid | 8 | 1 | 0.1 |
| Rifampicin | 5 | 1 | 0.1 |
| Miscellaneous | 152 | 22 | 2.0 |
|  |  |  |  |
| **Antifungal agent** |  |  |  |
| Anidulafungin | 475 | 67 | 75.2 |
| Fluconazole | 128 | 18 | 20.3 |
| Caspofungin | 14 | 2 | 2.2 |
| Amphotericin B | 13 | 2 | 2.1 |
| Posakonazole | 2 | 0 | 0.3 |

# DOT= days of therapy, PD=patient days, Relative use (%)= percent of use of antibiotic agents respective antifungal agents.

## *Tertiary care ICU*

| **Antibacterial agent** | **DOT** | **DOT/ 1000 PD** | **Relative use (%)** |
| --- | --- | --- | --- |
| Cefotaxime | 3442 | 300 | 26.6 |
| Piperacillin and tazobactam | 2335 | 204 | 18.0 |
| Meropenem | 1546 | 135 | 12.0 |
| Cefuroxime | 844 | 74 | 6.5 |
| Erythromycin | 532 | 46 | 4.1 |
| Cloxacillin | 530 | 46 | 4.1 |
| Metronidazole | 522 | 46 | 4.0 |
| Tigecycline | 473 | 41 | 3.7 |
| Clindamycin | 447 | 39 | 3.5 |
| Trimethoprim and sulph. | 388 | 34 | 3.0 |
| Vancomycin | 315 | 27 | 2.4 |
| Ciprofloxacin | 292 | 25 | 2.3 |
| Moxifloxacin | 322 | 28 | 2.5 |
| Penicillin G | 196 | 17 | 1.5 |
| Levofloxacin | 193 | 17 | 1.5 |
| Azitromycin | 130 | 11 | 1.0 |
| Linezolid | 92 | 8 | 0.7 |
| Ampicillin | 94 | 8 | 0.7 |
| Imipenem and cilastatin | 38 | 3 | 0.3 |
| Ceftriaxone | 31 | 3 | 0.2 |
| Ceftazidime and avibactam | 30 | 3 | 0.2 |
| Gentamicin | 18 | 2 | 0.1 |
| Rifampicine | 15 | 1 | 0.1 |
| Ertapeneme | 14 | 1 | 0.1 |
| Ceftolozan and tazobactam | 12 | 1 | 0.1 |
| Miscellaneous | 86 | 7 | 0.7 |
|  |  |  |  |
| **Antifungal agents** |  |  |  |
| Anidulafungin | 1256 | 110 | 82.2 |
| Fluconazole | 141 | 12 | 9.2 |
| Caspofungin | 61 | 5 | 4.0 |
| Posakonazole | 42 | 4 | 2.7 |
| Amphotericin B | 28 | 2 | 1.8 |

# DOT= days of therapy, PD=patient days, Relative use (%)= percent of use of antibiotic agents respective antifungal agents.
