# Supplementary material for: Automated surveillance of antimicrobial consumption in intensive care, northern Sweden: an observational case study
Source: Antimicrob Resist Infect Control. 2024 Jun 18;13:67. doi: 10.1186/s13756-024-01424-2 (PMC11186282; doi:10.1186/s13756-024-01424-2)
Supplement: Supplementary file 6 — Additional file 6. [file 13756_2024_1424_MOESM6_ESM.docx]

**Additional file 6**

**Antimicrobial consumption by the metrics DOT, administered and pharmacy dispensing DDD in the ICUs aggregated, by quarters 2018-2021.**

**
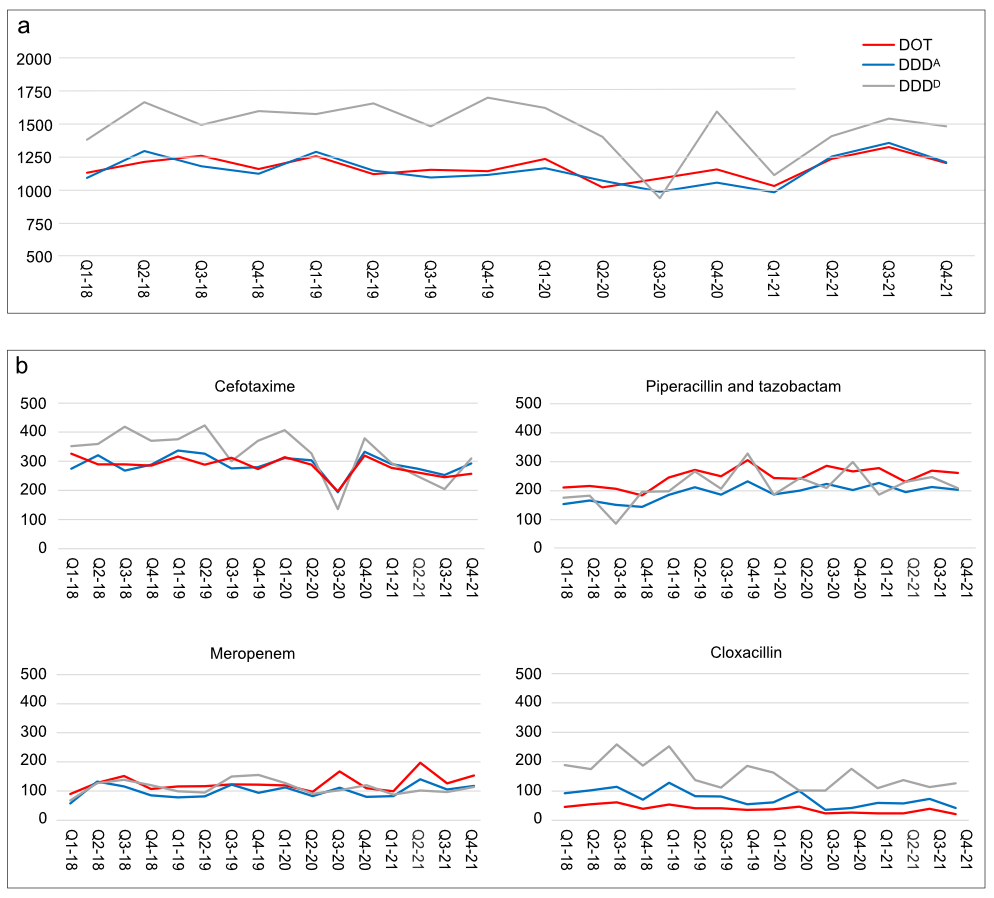
**

Additional file 6. Antimicrobial consumption by days of therapy (DOT), defined daily dose based on data from registered administrations (DDDᴬ) and based on pharmacy dispensing data (DDDᴰ) per 1000 patient days by quarters in the ICUs from 2018-2021. Panel a) total antimicrobial consumption, and panel b) four frequently used antibacterial agents.
