# Supplementary material for: Automated surveillance of antimicrobial consumption in intensive care, northern Sweden: an observational case study
Source: Antimicrob Resist Infect Control. 2024 Jun 18;13:67. doi: 10.1186/s13756-024-01424-2 (PMC11186282; doi:10.1186/s13756-024-01424-2)
Supplement: Supplementary file 7 — Additional file 7. [file 13756_2024_1424_MOESM7_ESM.docx]

**Additional file 7**

***Antimicrobial consumption by the metrics DOT, DDD based on registered administrations, and DDD based on dispensing data by ICU care level 2018-2021.***

| **Secondary care ICUs** | | | | | | | | | |
| --- | --- | --- | --- | --- | --- | --- | --- | --- | --- |
| **Antibiotic class** | **DOT** | **DOT/1000 PD** | **Relative consumption in DOT (%)** | **DDDᴬ** | **DDDᴬ/1000 PD** | **Relative consumption in DDD^A^ (%)** | **DDDᴰ** | **DDDᴰ/1000 PD** | **Relative consumption in DDD^B^ (%)** |
| Cephalosporins 2nd and 3rd generations | 1880 | 266 | 25 | 1838 | 263 | 28 | 2189 | 310 | 20 |
| Penicillin + β-lactamase inhibitor | 2227 | 315 | 30 | 1694 | 242 | 26 | 2176 | 308 | 20 |
| Carbapenems | 799 | 113 | 11 | 582 | 83 | 9 | 843 | 119 | 8 |
| Fluoroquinolones | 589 | 83 | 8 | 611 | 87 | 9 | 894 | 126 | 8 |
| Macrolides | 668 | 95 | 9 | 535 | 76 | 8 | 932 | 132 | 8 |
| Beta-lactamase resistant penicillin | 162 | 23 | 2 | 370 | 53 | 6 | 2070 | 293 | 19 |
| Imidazoles | 259 | 37 | 3 | 139 | 20 | 2 | 384 | 54 | 3 |
| Tetracyclines | 143 | 20 | 2 | 135 | 19 | 2 | 215 | 30 | 2 |
| Lincosamides | 275 | 39 | 4 | 225 | 32 | 3 | 311 | 44 | 3 |
| Glycopeptides | 133 | 19 | 2 | 153 | 22 | 2 | 189 | 27 | 2 |
| Other | 384 | 54 | 5 | 266 | 38 | 4 | 846 | 120 | 8 |

DOT = days of therapy, DDD = defined daily dose, PD = patient days, A = based on drug administrations to the patient, D = based on dispensing data, (%) indicates the proportion of the total antimicrobial consumption by each metric.

| **Tertiary care ICU** | | | | | | | | | |
| --- | --- | --- | --- | --- | --- | --- | --- | --- | --- |
| **Antibiotic class** | **DOT** | **DOT/1000 PD** | **Relative consumption in DOT (%)** | **DDDᴬ** | **DDDᴬ/1000 PD** | **Relative consumption in DDD^A^ (%)** | **DDDᴰ** | **DDDᴰ/1000 PD** | **Relative consumption in DDD^B^ (%)** |
| Cephalosporins 2nd and 3rd generations | 4322 | 377 | 35 | 4658 | 406 | 38 | 4876 | 400 | 39 |
| Penicillin + β-lactamase inhibitor | 2335 | 204 | 19 | 1869 | 162 | 15 | 1895 | 155 | 15 |
| Carbapenems | 1598 | 139 | 13 | 1281 | 112 | 10 | 1278 | 105 | 10 |
| Fluoroquinolones | 807 | 70 | 7 | 775 | 68 | 6 | 777 | 64 | 6 |
| Macrolides | 662 | 58 | 5 | 495 | 43 | 4 | 774 | 64 | 6 |
| Beta-lactamase resistant penicillin | 530 | 46 | 4 | 999 | 87 | 8 | 710 | 58 | 6 |
| Imidazoles | 522 | 46 | 4 | 395 | 32 | 3 | 410 | 34 | 3 |
| Tetracyclines | 473 | 41 | 4 | 450 | 37 | 4 | 485 | 40 | 4 |
| Lincosamides | 447 | 39 | 4 | 351 | 29 | 3 | 398 | 33 | 3 |
| Glycopeptides | 315 | 27 | 3 | 339 | 16 | 3 | 378 | 31 | 3 |
| Other | 272 | 24 | 2 | 526 | 46 | 4 | 655 | 57 | 5 |

DOT = days of therapy, DDD = defined daily dose, PD = patient days, A = based on drug administrations to the patient, D = based on dispensing data, (%) indicates the proportion of the total antimicrobial consumption by each metric.
